# Lithium Attenuates TGF-**β**_1_-Induced Fibroblasts to Myofibroblasts Transition in Bronchial Fibroblasts Derived from Asthmatic Patients

**DOI:** 10.1155/2012/206109

**Published:** 2012-09-03

**Authors:** Marta Michalik, Katarzyna Anna Wójcik, Bogdan Jakieła, Katarzyna Szpak, Małgorzata Pierzchalska, Marek Sanak, Zbigniew Madeja, Jarosław Czyż

**Affiliations:** ^1^Department of Cell Biology, Faculty of Biochemistry, Biophysics and Biotechnology, Jagiellonian University, Gronostajowa 7, 30-387 Kraków, Poland; ^2^Department of Medicine, Jagiellonian University Medical School, Skawińska 8, 30-031 Kraków, Poland; ^3^Department of Food Biotechnology, Faculty of Food Technology, University of Agriculture, Balicka 122, 31-149 Kraków, Poland

## Abstract

Bronchial asthma is a chronic disorder accompanied by phenotypic transitions of bronchial epithelial cells, smooth muscle cells, and fibroblasts. Human bronchial fibroblasts (HBFs) derived from patients with diagnosed asthma display predestination towards TGF-**β**-induced phenotypic switches. Since the interference between TGF-**β** and GSK-3**β** signaling contributes to pathophysiology of chronic lung diseases, we investigated the effect of lithium, a nonspecific GSK-3**β** inhibitor, on TGF-**β**_1_-induced fibroblast to myofibroblast transition (FMT) in HBF and found that the inhibition of GSK-3**β** attenuates TGF-**β**_1_-induced FMT in HBF populations derived from asthmatic but not healthy donors. Cytoplasmically sequestrated **β**-catenin, abundant in TGF-**β**_1_/LiCl-stimulated asthmatic HBFs, most likely interacts with and inhibits the nuclear accumulation and signal transduction of Smad proteins. These data indicate that the specific cellular context determines FMT-related responses of HBFs to factors interfering with the TGF-**β** signaling pathway. They may also provide a mechanistic explanation for epidemiological data revealing coincidental remission of asthmatic syndromes and their recurrence upon the discontinuation of lithium therapy in certain psychiatric diseases.

## 1. Introduction 

Bronchial asthma is a chronic disorder characterized by airway inflammation, remodeling of the airways, and their hyperresponsiveness to environmental stimuli. It is accompanied by thickening of the bronchial walls, epithelial damage, subepithelial fibrosis, and the increased deposition of extracellular matrix proteins. The bulk of these changes results from the phenotypic transitions of bronchial epithelial cells, smooth muscle cells, and fibroblasts [1, 2]. In general, phenotypic shifts, such as epithelial-mesenchymal transition (EMT) and fibroblast-myofibroblast transition (FMT), play a crucial role during various physiological and pathological processes [3–5]. In particular, erroneous function of myofibroblasts, representing the contractile subset of fibroblasts which have undergone FMT, was found to be crucial for the development of asthma [6, 7]. FMT, which is also involved in wound contraction and participates in the development of fibrotic changes in several tissues [8–10], can be induced by a range of proinflammatory cytokines, in particular by factors belonging to the transforming growth factor *β* (TGF-*β*) family [11, 12]. On the other hand, an increase in the local concentration of TGF-*β* was observed in bronchoalveolar lavage fluid sampled from asthmatic patients [13], and prolonged exposure to TGF-*β* effectively induced FMT in HBFs *in vitro* models [14]. 

It is generally accepted that TGF-*β* regulates transcription of profibrotic genes, including cell adhesion molecules and extracellular matrix proteins [15, 16]. The canonical TGF-*β* signaling pathway depends on the phosphorylation of R-Smad proteins (Smad2 and 3) by activated TGF-*β* receptor, their interaction with Smad4, and accumulation in the nuclei [17, 18]. This process is antagonized by I-Smads (6 and 7). TGF-*β* signaling activity is modulated by other signaling systems, such as the Wnt-dependent pathway [19, 20], which has also been implicated in the pathophysiology of lung cancers, asthma, and lung fibrotic disease development [21–25]. TGF-*β* was shown to induce FMT in human lung fibroblast populations [26]; however, it attenuated the allergic inflammation and asthmatic symptoms in an ovalbumin-induced mouse model of asthma [27]. Activation of Wnt pathway results in the inhibition of GSK-3*β* (glycogen synthase kinase 3 beta) and subsequent cytoplasmic accumulation of *β*-catenin. It forms complexes with transcription factors of the Lef and TCF family activating the expression of Wnt-responsive genes [28]. A range of interactions between Wnt and TGF-*β* effectors have been identified, which conceivably account for synergic effects of TGF-*β* and Wnt signaling observed *in vitro* during FMT in mouse embryonic fibroblast populations [25]. For instance, MAPK can phosphorylate both R-Smads and GSK-3*β* [25, 26], while *β*-catenin was found to interact with I-Smads [29]. These interactions may also underline cellular context-dependent role of GSK-3*β* activity in asthma development. 

Lithium is a nonspecific GSK-3*β* inhibitor widely used in the therapy of bipolar affective disorder and other psychiatric diseases [30]. Interestingly, lithium treatment of schizoaffective disorders coincided with the remission of asthmatic symptoms [31], whereas the recurrence of symptomatic asthma was observed after discontinuation of lithium therapy [32]. Moreover, treatment with lithium led to a reduction in bronchial hyperresponsiveness in asthmatic patients [33]. We have previously shown that HBFs derived from patients with asthma displayed more pronounced FMT in response to TGF-*β* in comparison with nonasthmatic counterparts. This indicates susceptibility towards a TGF-*β*-induced phenotypic switch, an event crucial for the asthmatic process [14, 34]. In the current study, we aimed to elucidate the role of lithium compounds in TGF-*β*_1_-induced FMT in HBF populations and the mechanisms responsible for this process. We demonstrate inhibition of GSK-3*β* activity by lithium attenuated TGF-*β*_1_-induced FMT in HBF populations derived from asthmatic donors. This effect correlated with deficient nuclear localization of *β*-catenin and p-Smad2 in TGF-*β*_1_/LiCl-stimulated HBFs.

## 2. Materials and Methods

### 2.1. Isolation of Human Bronchial Fibroblasts (HBFs)

Bronchial biopsies were obtained from 11 patients with bronchial asthma (AS) and 11 non-asthmatic subjects (NA) during fiberoptic bronchoscopy performed in the Department of Medicine (Jagiellonian University Medical College). Primary HBF were isolated from bronchial biopsies as described previously [14, 34]. The first group consisted of patients with moderate to severe bronchial asthma (AS group) with a mean age of 43.1 ± 9.5 years (females 64%), the average duration of the disease was 11.3 ± 9.4 years, and decrease in the first second forced expiratory volume (FEV1: 78.4 ± 22.4% of predicted). The second group comprised 11 subjects in whom diagnostic bronchoscopy ruled out any serious airway pathology, including asthma, chronic inflammatory lung disease, or cancer (nonasthmatics; NA group), with mean age of 58.7 ± 7.8 years (females 36%) and normal lung function tests (FEV1: 97.3 ± 12.8% of predicted). The study was approved by the Jagiellonian University's Ethics Committee (KBET/362/B/2003), and informed consent was obtained from all study participants. 

### 2.2. Cell Culture Protocols

HBFs were cultured in DMEM with 10% fetal bovine serum (FBS) at 37°C in a humidified atmosphere with 5% CO_2_ and used between 5 and 15 passages. For experiments, cells were plated at a density of 5000 cells/cm^2^ and cultured in serum-free DMEM supplemented with 0.1% bovine serum albumin (BSA; Sigma-Aldrich, St. Louis, MO, USA). When indicated, human recombinant TGF-*β*_1_ (BD Biosciences, Franklin Lakes, NJ, USA), lithium chloride (LiCl; Sigma-Aldrich) or their cocktail, and GSK-3*β* inhibitor XII, TWS119 (Calbiochem, La Jolla, CA, USA) were administered 24 h after cell seeding. Lithium chloride was administered at the concentration of 10 mM, which remained in a range of serum concentrations observed in patients subjected to the chronic therapy of bipolar affective disorder [35, 36].

### 2.3. Immunofluorescence Staining

Myofibroblasts were identified by immunodetection of *α*-SMA as described previously [34]. In brief, cells growing on glass coverslips were fixed in 3.7% paraformaldehyde, permeabilised in 0.1% Triton X-100, blocked with 1% BSA, and incubated with a mouse monoclonal antibody against human *α*-SMA (clone 1A4, Sigma-Aldrich) and Alexa Fluor 488 goat anti-mouse IgG (clone A11001, Sigma-Aldrich). Visualization of specimens mounted in polyvinyl alcohol (Mowiol; Sigma-Aldrich) was performed with a Leica DM IRE2 microscope equipped with 40x, NA-1.25 HCX Plan Apo objective, Leica DC350FX camera, and Leica FW4000 software. A similar protocol was used for immunodetection of other cellular antigens including TGF-*β* receptor II (with a mouse monoclonal antibody against TGF-*β*-RII, clone MM0056-4F14, Abcam, Cambridge, UK), *β*-catenin (with a mouse monoclonal antibody against *β*-catenin, Sigma-Aldrich) or p-Smad (with a rabbit polyclonal antibody against Smad2, phospho S467, Abcam), and compatible secondary antibodies conjugated with Alexa Fluor 488 or Alexa Fluor 546 (all from Invitrogen, Carlsbad, CA, USA). When indicated, nuclei were stained with Hoechst 33342 (Sigma-Aldrich). 

### 2.4. Flow Cytometry Analyses

For the analyses of TGF-*β* receptor II expression on the surface of HBF, confluent cell monolayers were nonenzymatically detached using the cell dissociation solution (Sigma-Aldrich) according to the manufacturer's protocol, centrifuged, fixed and permeabilized with Cytofix/Cytoperm Kit (BD Biosciences), and stained with primary mouse monoclonal anti-TGF-*β* Receptor II antibody (Abcam). After washing with Perm-Wash buffer (BD Biosciences), cell suspensions were stained with secondary FITC-conjugated goat anti-mouse IgG (Sigma-Aldrich) and analysed by flow cytometry (Coulter EPICS XL, Beckman Coulter, Fullerton, CA). Results were expressed as a ratio of mean fluorescent intensity (MFI) of TGF-*β*-RII-specific staining to MFI of the corresponding goat-IgG2a isotype control sample (expression index).

### 2.5. Cell Fractionation and Immunoblotting

HBFs cultured in 6 cm Petri dishes for 7 days (until confluency) in control conditions or stimulated with TGF-*β*_1_ (5 ng/mL), LiCl (10 mM) and their mixture (TGF-*β*_1_/LiCl), or TWS119 (5 *μ*M) were washed, harvested using a cell scraper, centrifuged, and dissolved in lysis buffer (50 mM Tris-HCl pH 7.4, 150 mM NaCl, 2% TritonX-100, 0.02% NaN_3_ with the addition of a proteinase inhibitor). Alternatively, total protein was isolated using M-PER buffer (Pierce, Rockford, IL, USA) with protease and phosphatase inhibitors (Sigma-Aldrich), and the nuclear fraction was isolated using NEB-B buffer (29 mM HEPES pH 7.9; 0.4 M NaCl; 1 mM EDTA; 1 mM EGTA). In both cases, the protein content was determined with the Bradford method. Cellular proteins were separated on 15% SDS-polyacrylamide gels and transferred to nitrocellulose membranes. After blocking in PBS-T (0.1% Tween 80 in PBS containing 5% skimmed milk), membranes were incubated with mouse monoclonal antibodies (all from Sigma-Aldrich), anti-*α*-SMA (1 : 1000), anti-*β*-catenin (1 : 2000), anti-*α*-tubulin (1 : 1000), anti-GAPDH (1 : 10000), and rabbit polyclonal antibodies anti-human Retinoblastoma-associated protein 46 (RbAp46, 1 : 2000). After washing, membranes were incubated for 1 hour with horseradish peroxidase-conjugated anti-mouse IgG (1 : 3000, Invitrogen) or anti-rabbit IgG (1 : 3000, Invitrogen) antibodies, treated with the chemiluminescent reagent Super Signal West Pico Substrate (Pierce, Rockford, IL) and exposed to Kodak X-Omat film (Sigma-Aldrich). 3 independent experiments were performed for each condition. 

## 3. Results

### 3.1. Reactivity of HBF Derived from Asthmatic and Nonasthmatic Patients to TGF-*β*_1_

**Figure 1:**
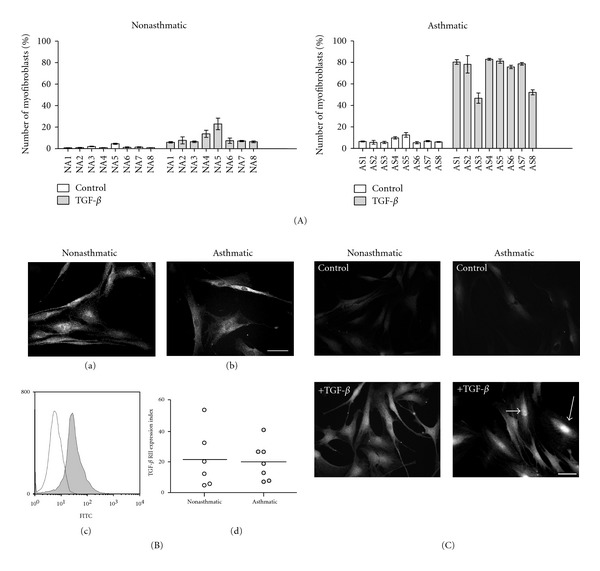
Phenotypic shifts in nonasthmatic and asthmatic HBF populations induced by TGF-*β*_1_. Administration of TGF-*β*_1_ resulted in a prominent FMT in AS HBF populations, illustrated by relatively high fractions of *α*-SMA-positive cells (A). This effect is independent of TGF-*β* RII levels (B) but correlates with nuclear accumulation of p-Smad2 (C). (A) HBFs from NA (*n* = 8) and AS (*n* = 8) were cultured in DMEM with 10% FCS for 24 hours, and then in serum-free conditions with or without 5 ng/mL TGF-*β*_1_ for 7 days, and fractions of *α*-SMA-positive cells were detected. Each column represents the percentage of myofibroblasts for each individual HBF culture (±SE). In the AS group, the percentage of positive cells was significantly higher than in the NA group in TGF-*β*_1_-treated cultures (Kruskal-Wallis nonparametric ANOVA, *P < 0.05). (B) For the estimation of TGF-*β* RII levels, NA and AS HBFs were cultured in DMEM with 10% FCS for 24 hours, immunostained for TGF-*β* RII, and analyzed by both fluorescence microscopy and FACS. Representative fluorescence microscopy images (bar = 50 *μ*m) illustrate a similar pattern of expression of TGF-*β* RII in HBF from NA (a) and AS (b) cultures. Representative histogram showing the specificity of TGF-*β* RII staining in comparison with isotype control (c). TGF-*β* RII expression levels were similar in both studied groups. Each point of the graph (d) represents data obtained from a single cell population (NA or AS) and horizontal bars represent the mean values. (C) TGF-*β*_1_-induced p-Smad2 translocation to the nucleus in HBFs. Fibroblasts from NA and AS were cultured in serum-free conditions for 24 hours and then with (+TGF-*β*) or without (control) TGF-*β*_1_ (5 ng/mL), next cells were immunostained for p-Smad2 and photographed with fluorescence microscopy. After 30 minutes of treatment with TGF-*β*_1_, nuclear accumulation of p-Smad2 was detectable, and was more pronounced in AS HBFs (arrows). Bar = 50 *μ*m.

We have previously demonstrated that HBFs derived from patients with bronchial asthma display inherent features *in vitro* that facilitate FMT in response to prolonged incubation with TGF-*β*_1_ [14, 34]. Here, we aimed to investigate the interference of lithium with TGF-*β*_1_-induced FMT in HBF populations derived from asthmatic patients (AS, *n* = 8) and non-asthmatic subjects (NA, *n* = 8). Therefore, we first quantified *α*-SMA-positive cells in HBF populations which had undergone long-term TGF-*β*_1_ stimulation. We have found a considerably higher fraction of *α*-SMA expressing HBFs in AS as compared to NA populations (Figure 1(A)) which indicates their propensity towards a TGF-*β*_1_-induced phenotypic switch. This effect was independent of the total density of TGF-*β* receptor as both immunofluorescence microscopy and flow cytometry analyses did not reveal any significant differences in expression levels between AS and NA patients (Figure 1(B)). Furthermore, cytoplasmic levels of p-Smad2 protein, an effector of TGF-*β* signalling, and its nuclear localisation were increased in TGF-*β*_1_-treated HBF populations, both in NA and AS groups. However, we observed a slightly more pronounced nuclear accumulation of p-Smad2 in TGF-*β*_1_-stimulated asthmatic HBF populations (Figure 1(C)). These results suggest that mechanisms downstream of the TGF-*β* receptor which modulate nuclear transport of Smad protein(s) determine the differences in TGF-*β*_1_ reactivity between HBF derived from asthmatic and non-asthmatic patients. 

### 3.2. Lithium Attenuates TGF-*β*_1_-Stimulated FMT in Asthmatic HBF Cultures

**Figure 2:**
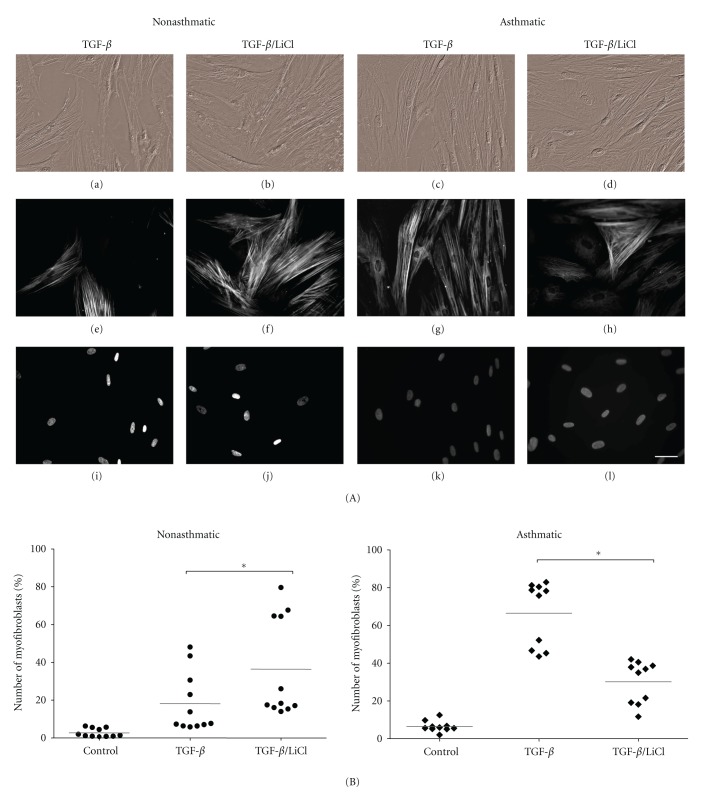
The effect of LiCl on TGF-*β*_1_-stimulated FMT in non-asthmatic and asthmatic HBFs. HBFs from NA (*n* = 11) and AS (*n* = 10) groups were cultured in DMEM supplemented with 0.1% BSA and TGF-*β*_1_ (5 ng/mL), LiCl (10 mM), or TGF-*β*_1_/LiCl (A, B). (A) Representative images of HBF from NA and AS culture: Nomarski interference contrast microscopic images (a–d), immunofluorescent staining of *α*-SMA (e–h), and Hoechst staining of cell nuclei (i–l). Bar = 50 *μ*m. (B) Graphs showing the percentage of myofibroblasts after 7-day culture of HBFs. Each point represents a single HBF culture derived from NA and AS groups (the means of at least three separate experiments for each culture are presented, in the one experimental point at least 100 cells were counted; only HBFs with *α*-SMA incorporated into stress fibres were considered as *α*-SMA positive, i.e, myofibroblasts). Bars on graphs represent mean values. The significance of differences between TGF-*β*_1_-stimulated HBFs without LiCl and with LiCl was determined by applying Kruskal-Wallis non-parametric ANOVA, *P < 0.05.

It has been found that GSK-3*β*-dependent pathways interact with TGF-*β* signaling in a variety of cellular systems [37, 38]. Similarly, lithium which at millimolar concentrations displays GSK-3*β* inhibiting activity, has been shown to interfere with the asthmatic process [32, 33]. Application of the experimental system based on quantification of FMT in HBF populations derived from AS and NA patients enabled us to elucidate the effect of cellular context on FMT-related cell reactivity to lithium (Figure 2). We have not observed any significant differences in the viability and percentage of myofibroblasts between untreated (control) and treated by LiCl alone in HBF cultures, regardless of the source of cells (data not shown). In contrast to TGF-*β*_1_-treated HBF from the NA group, which reacted to the administration of lithium with the induction of FMT, AS HBFs treated with TGF-*β*_1_/LiCl displayed a decrease in the fraction of myofibroblasts compared to the cells stimulated with TGF-*β*_1_ alone (43 to 85% of *α*-SMA positive cells in the presence of TGF-*β*_1_ compared to 15–42% in TGF-*β*_1_/LiCl mixture; Figure 2(B)). These data indicate that increased susceptibility of AS HBFs to TGF-*β*_1_-induced FMT is associated with a differential response to LiCl as compared to NA and suggest the presence of a possible disturbance in crosstalk between signalling pathways regulated by TGF-*β*_1_ and GSK-3*β*.

**Figure 3:**
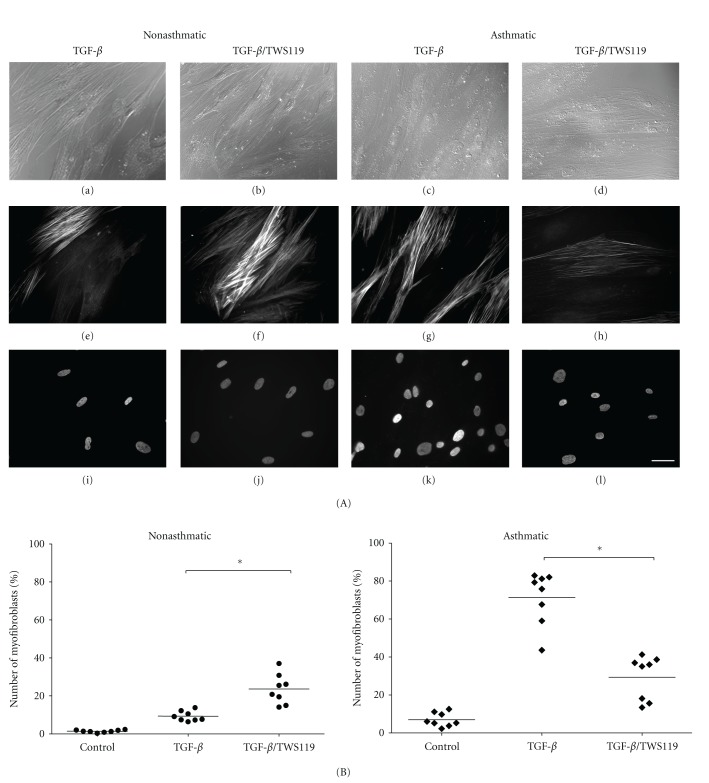
The effect of inhibition of GSK-3*β* on TGF-*β*_1_-stimulated FMT in nonasthmatic and asthmatic HBFs. HBFs from non-asthmatics (*n* = 8) and asthmatics (*n* = 8) were cultured in DMEM supplemented with 0.1% BSA and TGF-*β*_1_ (5 ng/mL), TWS119 (10 *μ*M), or TGF-*β*_1_/TWS119 (A, B). (A) Representative images of HBF from NA and AS culture: Nomarski interference contrast microscopic images (a–d), immunofluorescent staining of *α*-SMA (e–h), and Hoechst staining of cell nuclei (i–l). Bar = 50 *μ*m. (B) Graphs showing the percentage of myofibroblasts after 7-day culture of HBFs. Each point represents a single HBF culture from NA and AS groups (the means of at least three separate experiments for each culture are presented, in the one experimental point, at least 100 cells were counted). Bars on graphs represent mean values. The significance of differences between TGF-*β*_1_-stimulated HBFs without TWS119 and with the inhibitor was determined by applying Kruskal-Wallis non-parametric ANOVA, *P < 0.05.

**Figure 4:**
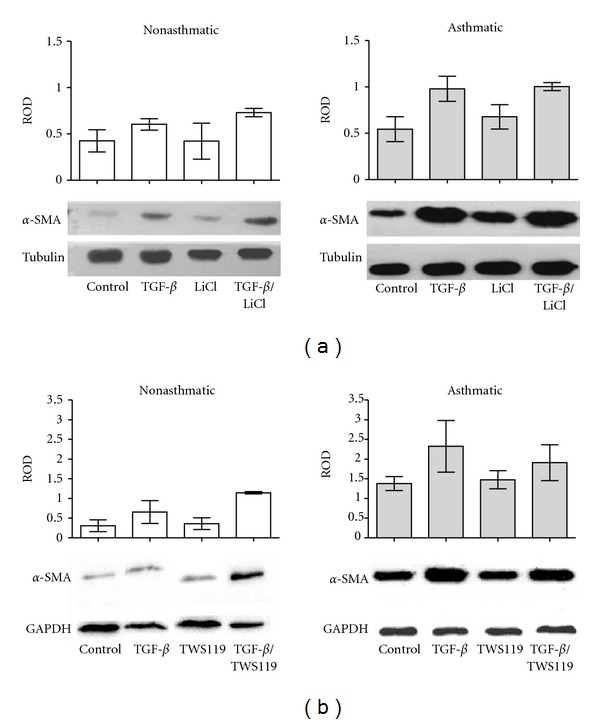
The effect of inhibition of GSK-3*β* on TGF-*β*_1_-stimulated *α*-SMA levels in nonasthmatic and asthmatic HBFs. HBFs from NA and AS groups were cultured for 7 days in DMEM supplemented with 0.1% BSA (control), or with the same medium with addition of TGF-*β*_1_ (5 ng/mL), LiCl (10 mM), or TGF-*β*_1_/LiCl (a) or with the same control medium supplemented with TGF-*β*_1_ (5 ng/mL), TWS119 (10 *μ*M) or TGF-*β*_1_/TWS119 (b). Protein extracts (10 *μ*g of protein per lane) were subjected to SDS-PAGE electrophoresis, and *α*-SMA protein/*α*-tubulin (a) or *α*-SMA protein/GAPDH (b) was detected by immunoblotting. Responses were quantified by densitometry and normalized to the expression of *α*-tubulin or GAPDH, respectively. Data represents relative optical density mean of at least 3 experiments (ROD ± SE). Representative immunoblots are shown under the graphs.

To check whether the attenuation of TGF-*β*_1_-induced FMT in AS HBF populations treated with lithium specifically results from its inhibitory effect on GSK-3*β* activity, we further treated the cells with TWS-119, a specific GSK-3*β* inhibitor [39]. Its effect on the number of myofibroblasts in TGF-*β*_1_-treated NA and AS HBF populations was found to be similar to that exerted by lithium (Figure 3) indicating the involvement of GSK-3*β* activity in the observed phenomena. Moreover, in NA HBF populations treated with TGF-*β*_1_/LiCl or TGF-*β*_1_/TWS-119, the percentage of *α*-SMA-positive cells corresponded to *α*-SMA protein levels (compare Figure 2 to Figure 4(a) and Figure 3 to Figure 4(b)). On the other hand, different results were obtained for AS HBF populations. Despite lower numbers of *α*-SMA-positive cells, the same amounts of *α*-SMA were observed in TGF-*β*_1_ and in TGF-*β*_1_/LiCl (Figure 4(a)). This effect was not observed in TGF-*β*_1_/TWS-119-treated AS HBF populations (Figure 4(b)). This can be explained in terms of GSK-3*β*-unspecific induction of “supermaturation” and hypertrophy of a subset of AS HBF subjected to TGF-*β*_1_/LiCl treatment. Such a sparse subpopulation characterised by a high degree of spreading and intense *α*-SMA-specific staining was found in AS HBF populations treated with the TGF-*β*_1_/LiCl mixture (data not shown). Moreover, it should be noticed that the effect of GSK-3*β* inhibition on FMT is much more striking when comparing the percentage of myofibroblasts to the *α*-SMA protein levels. The reason of it could be that not all expressed *α*-SMA is incorporated into stress fibers which are the marker of myofibroblasts. 

Altogether, these data indicate that GSK-3*β* participates in the attenuating effect of lithium on TGF-*β*_1_-induced FMT in AS HBF populations; however, lithium may exert a GSK-3*β*-unspecific effect on the maturation of myofibroblasts [40].

### 3.3. Lithium Reduces TGF-*β*_1_-Stimulated Nuclear Translocation of p-Smad in Asthmatic HBFs

**Figure 5:**
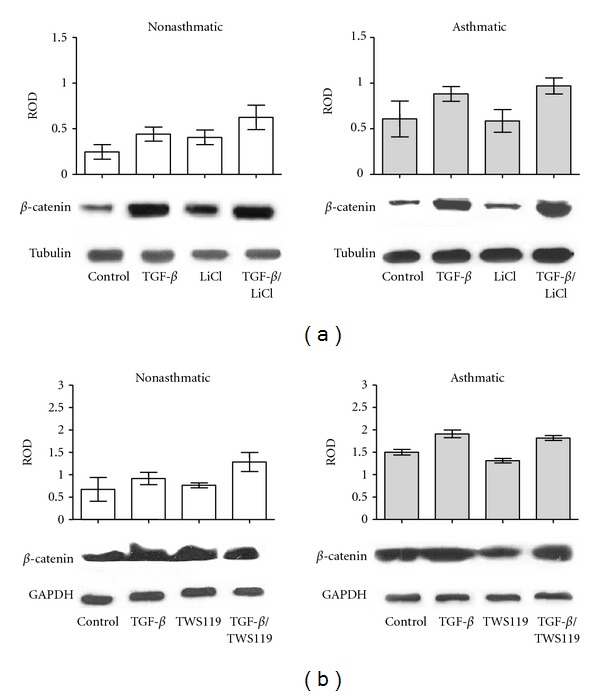
The effect of inhibition of GSK-3*β* on *β*-catenin expression in control and TGF-*β*_1_-stimulated HBFs. HBFs from NA and AS populations were cultured in DMEM supplemented with 0.1% BSA (control) and with the same medium supplemented with TGF-*β*_1_ (5 ng/mL), LiCl (10 mM), or TGF-*β*_1_/LiCl (a) or in DMEM supplemented with 0.1% BSA (control) and with the same medium supplemented with TGF-*β*_1_ (5 ng/mL), TWS119 (10 *μ*M), or TGF-*β*_1_/TWS119 (b) for 7 days. Total protein extracts (30 *μ*g of protein per lane) were subjected to SDS-PAGE electrophoresis, and *β*-catenin/*α*-tubulin protein (a) or *β*-catenin/GAPDH protein (b) was detected by immunoblotting. The graphs in (a) and (b) represent results of densitometric analysis of immunoblots. The amount of *β*-catenin was normalized to the expression of *α*-tubulin or GAPDH, respectively. Representative immunoblots from 3 independent experiments are shown under the graphs.

**Figure 6:**
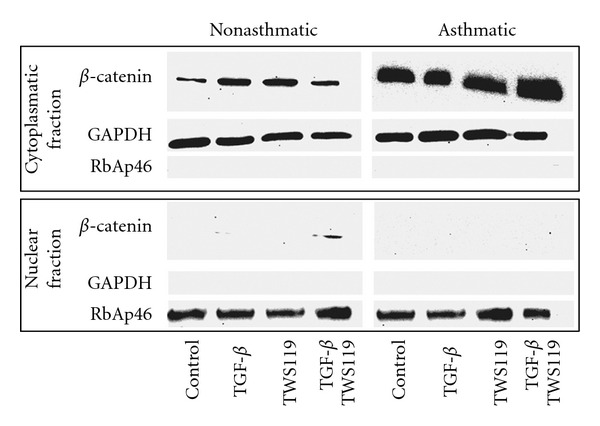
Nuclear localization of *β*-catenin is inhibited in asthmatic fibroblasts. HBFs from NA and AS populations were cultured in DMEM supplemented with 0.1% BSA (control) or supplemented with TGF-*β*_1_ (5 ng/mL), TWS119 (10 *μ*M), or TGF-*β*/TWS119 for 7 days. Nuclear and cytoplasmic fractionswere isolated, and then the protein extracts were subjected to SDS-PAGE electrophoresis. *β*-catenin protein was detected by immunoblotting. The purity of the cytoplasmic and nuclear fractions was tested by detection of GAPDH and RbAp46. Note the increased amount of *β*-catenin in AS HBF in comparison to NA cells and that in the nucleus immunodetectable *β*-catenin was only found in NA fibroblasts after treatment with TGF-*β*/TWS119 mixture. Representative immunoblots from 3 independent experiments are shown.

**Figure 7:**
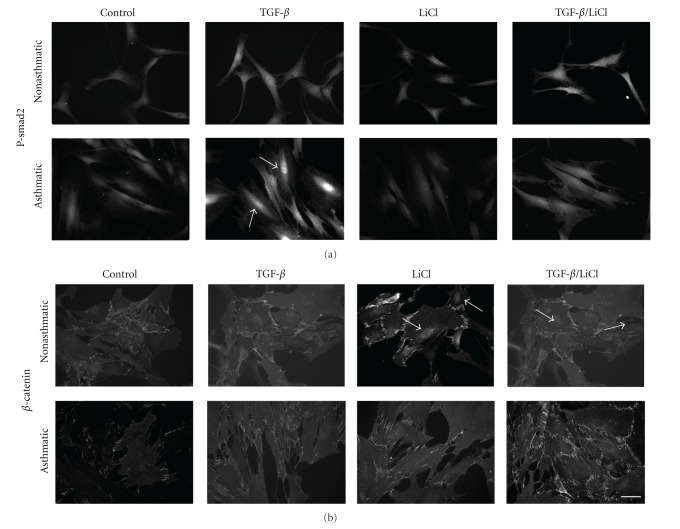
P-Smad and *β*-catenin localization in non-asthmatic and asthmatic HBFs. HBFs from NA and AS were cultured in DMEM with 10% FCS for 24 hours, then in DMEM with 0.1% BSA for 24 hours and with the same medium supplemented with TGF-*β*_1_ (5 ng/mL), LiCl (10 mM)s or TGF-*β*_1_/LiCl for 1 hour. Next, cells were immunostained for p-Smad2 (a) or *β*-catenin (b) and photographed with fluorescence microscopy. Arrows point to intensively stained nuclei. Bar = 50 *μ*m.

Inhibition of GSK-3*β* activity results, among others, in the hypophosphorylation of *β*-catenin and its nuclear translocation leading to the activation of gene expression^.^[41]. Therefore, we further focused on the effect of lithium and TWS119 on *β*-catenin levels in NA and AS HBF populations stimulated by TGF-*β*_1_. Interestingly, AS HBFs are characterized by a notably higher amount of total *β*-catenin (Figure 5) and *β*-catenin cytoplasmic fraction (Figure 6). TGF-*β*_1_ alone increased total *β*-catenin levels in both groups of HBF. Administration of TGF-*β*_1_/LiCl and TGF-*β*_1_/TWS119 resulted in the additional increase of the *β*-catenin level in NA populations, in contrast to AS cells (Figures 5(a) and 5(b)). On the other hand, inhibition of GSK-3*β* regardless of TGF-*β*_1_ treatment resulted in the nuclear accumulation of *β*-catenin in NA HBF, whereas this effect was not observed in AS HBF populations (Figures 6 and 7(b)). Thus, the impaired intracellular trafficking of *β*-catenin may account for differences in cell reactivity to TGF-*β*_1_ stimulation and FMT between NA and AS bronchial fibroblasts.

To further clarify the mechanism of the observed phenomenon, we elucidated the effect of the LiCl and TGF-*β*_1_/LiCl mixture on nuclear accumulation of p-Smad2, an event crucial for the activation of TGF-*β* signalling activity (Figure 7). Treatment with the TGF-*β*_1_ and TGF-*β*_1_/LiCl cocktail induced p-Smad2 nuclear accumulation in NA HBF populations but not in AS HBF populations. TGF-*β*_1_-induced nuclear translocation of p-Smad2 was significantly attenuated by lithium in AS HBF populations (Figure 7(a)). Interestingly, this effect correlated with the pattern of nuclear accumulation of *β*-catenin (Figure 7(b)). These data suggest a role for the interference of the *β*-catenin cytoplasmatic fraction with the function of p-Smad2 during FMT in AS HBF populations.

## 4. Discussion

Bronchial wall remodeling, a fundamental factor in the development of asthma, results from erroneous patterns of differentiation of bronchial cells, in particular fibroblasts [1, 2]. These changes are regulated by elevated production of proinflammatory cytokines, in particular TGF-*β*, in regions of epithelial damage as reflected in bronchoalveolar lavage fluid from asthmatics [13, 42]. FMT, a crucial event during bronchial wall remodeling and development of fibrotic changes observed in asthma, is supposed to depend on a concerted action of inflammatory mechanisms and inherent features of airway wall fibroblasts facilitating their phenotypic transitions in response to IL-4 [43] and TGF-*β* [14, 34]. Our previous [14] and current observations indicate that increased local concentrations of TGF-*β*, resulting from inflammation, and differences in the expression levels of TGF-*β* receptors are not necessarily implicated in bronchial wall remodeling during the asthma. In contrast, more pronounced differences in functional status of HBFs residing in healthy and asthmatic bronchi, for example, resulting from genetic patients' background or selection of clones epigenetically predisposed to FMT, can account for the differences in attitude towards FMT observed between HBFs derived from asthmatic and non-asthmatic patients. We show that inhibition of GSK-3*β* activity significantly attenuates TGF-*β*_1_-induced FMT in HBF derived from asthmatic patients, whilst an opposite effect was observed in HBF isolated from non-asthmatic subjects. Thus, specific molecular mechanisms involving the interactions between Smad proteins and effectors of GSK-3*β*-dependent signaling determine FMT-related responses of HBF isolated from asthmatic patients. 

The observed augmentation of TGF-*β*_1_-induced FMT by lithium in NA HBF populations, along with a lack of differences in TGF-*β* receptor expression levels between NA and AS HBFs, indicates the involvement of synergistic TGF-*β*/GSK-3*β* signaling interplay in phenotypic shifts during differentiation of bronchial fibroblasts. Such an induction of FMT-related processes by synergic activation of TGF-*β*/GSK-3*β* signaling was previously seen in mouse mesenchymal C3H10T1/2 cells [44], mouse embryonic fibroblasts [25], and pulmonary fibroblasts from individuals with chronic obstructive pulmonary disease [45]. The inhibition of GSK-3*β* is a key event during the activation of canonical Wnt pathway and leads to hypophosphorylation of *β*-catenin, its cytoplasmatic accumulation, subsequent nuclear translocation of *β*-catenin complexes with transcription factors [38], and induction of Wnt-responsive gene expression [24]. Our data, demonstrating a correlation between *α*-SMA expression, *β*-catenin levels, and nuclear translocation of *β*-catenin in the NA HBF populations treated with TGF-*β*_1_/LiCl and TGF-*β*_1_/TWS119 cocktails, reveals the involvement of the *β*-catenin-dependent pathway in phenotypic transitions of bronchial fibroblasts. These data are in line with the *in vitro* observations of similar interrelations in human lung [26] and skin fibroblast [46], and lung smooth muscle cell populations [47] and with the *in vivo* data on reduced bleomycin-induced pulmonary fibrosis upon *β*-catenin silencing in the murine model [48]. Notably, our study was focused on *α*-SMA expression as a marker of FMT; however, Wnt signaling controls the expression of a number of genes in lung cells, including fibronectin and collagen [49]. Importantly, we have recently shown that the inhibition of GSK-3*β* activity by TWS119 attenuates the secretion of CTGF by HBF derived from asthmatic patients [50]. 

On the other hand, attenuation of TGF-*β*_1_-induced FMT by lithium observed in HBF populations derived from asthmatic patients may indicate that inherent features facilitating FMT are accompanied by disturbed coherence of TGF-*β*_1_/GSK-3*β* signaling pathways. Actually, an induction of FMT by TGF-*β*_1_ coincided with an increased abundance of *β*-catenin in AS but not NA HBF populations. In contrast, high levels of *β*-catenin accompanied a relative decrease of *α*-SMA-positive cell numbers in TGF-*β*_1_/LiCl-treated AS HBF populations. Thus, the function of *β*-catenin during FMT may depend on the activity of GSK-3*β* modulated by lithium or TWS119. The specific inhibition of GSK-3*β* by TWS119 failed to induce the nuclear translocation of *β*-catenin in AS HBF populations. Therefore, the attenuating effect of lithium on FMT in AS HBF populations may result from disturbed intracellular *β*-catenin trafficking and its antagonistic effect on the function of downstream effectors of TGF-*β*_1_ [29, 51–53]. The phosphorylation of Smad proteins and their subsequent accumulation in the nuclei are key events during the activation of TGF-*β* signaling [17, 18]. Because we observed deficient nuclear accumulation of p-Smad2 in AS HBF populations treated with a TGF-*β*_1_/LiCl cocktail, it is conceivable that cytoplasmically sequestrated *β*-catenin may interact with Smad proteins inhibiting their nuclear accumulation and TGF-*β*-specific signal transduction. Elevated levels of cytoplasmatic *β*-catenin were previously demonstrated to inhibit p-Smad2 nuclear accumulation via the inhibition of iSMAD phosphorylation and degradation [54]. Therefore, further study is necessary to address the role of iSMAD proteins, in particular the possible involvement of their inhibitors, such as peptidyl-prolyl isomerases, in the observed differences between the lithium-evoked reactions of AS and NA HBFs [55]. 

Our finding of an inhibitory effect of lithium on TGF-*β*_1_-induced FMT in AS HBF populations provides a potential explanation for the attenuating effect of lithium on the ongoing asthmatic process, as well as coincidental remission of asthmatic symptoms and their recurrence upon discontinuation of lithium therapy in psychiatric diseases, as revealed by epidemiological studies [31–33]. However, it should be emphasized that numerous adverse effects of lithium have been reported [30]. Nonetheless, other drugs targeting the Wnt/*β*-catenin pathway were reported to be potentially effective in treating cancer [56] and tissue remodeling after injury [57]. Significant progress has recently been made in identifying such molecules and inventing new strategies of inhibition of Wnt signaling in therapeutic applications. 

Therefore, further research focused on the cell context-specific action of lithium or other Wnt pathway activators during the asthmatic process, and the mechanisms of interference of these compounds with phenotypic transitions of bronchial fibroblasts would help to delineate borders of future clinical application in acute asthma treatment.
